# Comparative performance analysis for abdominal phantom ROI detectability according to CT reconstruction algorithm: ADMIRE

**DOI:** 10.1002/acm2.12765

**Published:** 2019-11-15

**Authors:** Jun‐Bong Shin, Do‐Kun Yoon, Seongyong Pak, Yang‐Ho Kwon, Tae Suk Suh

**Affiliations:** ^1^ Department of Biomedical Engineering and Research Institute of Biomedical Engineering College of Medicine Catholic University of Korea Seoul Korea; ^2^ Siemens Healthineers Ltd. Seoul Korea

**Keywords:** abdomen phantom, ADMIRE, FBP, MDCT, SAFIRE

## Abstract

**Purpose:**

We compared and analyzed the detectability performance pertaining to an abdominal phantom including a region of interest (ROI) according to a computed tomography (CT) reconstruction algorithm.

**Methods:**

Three types of reconstruction algorithms (FBP, SAFIRE, and ADMIRE) were used to evaluate the detectability performance using the abdominal phantom (phantom size: 25 × 18 × 28 cm^3^). The vendor default settings for routine multi‐detector computed tomography abdominal scans were used. As the quantitative evaluation method, the contrast‐to‐noise ratio (CNR), difference in coefficient of variation (COV) with the normalization based on the FBP data, and the noise power spectrum (NPS) were measured.

**Results:**

The characteristic of the ADMIRE‐3 reconstructed image was higher than those of the FBP and SAFIRE‐3 reconstructed images. The CNR values of the SAFIRE and ADMIRE images were much higher than the corresponding values of the FBP images. The difference in COV values for the ADMIRE images was ~1.2 times lower than the corresponding values of the SAFIRE images.

**Conclusion:**

The comparative analysis of the abdominal phantom low‐contrast resolution differences for each CT exposure parameters showed that ADMIRE demonstrated better results than SAFIRE and FBP in terms of contrast, CNR, COV difference, and 1D NPS. This indicates that ADMIRE can provide a clearer observation even with the same number of contrast objects as compared to SAFIRE and FBP owing to its better contrast resolution in the central part of the contrast hole at low kV.

## 1 INTRODUCTION

Along with the development of computed tomography (CT) technology, the method of image reconstruction has developed dramatically. While analytical algorithms such as the commonly used filtered back projection (FBP) are based on only a single reconstruction, iterative algorithms are used to preserve the image noise while preserving the fine projection details through multiple reconstruction.^1^

The clinical field is actively performing CT examinations using the repetitive reconstruction algorithm. To this end, iterative reconstruction algorithms may allow a notable dose reduction as they facilitate a more precise modeling of the acquisition process.^2^, ^3^, ^4^, ^5^, ^6^, ^7^

In the advanced modeled iterative reconstruction algorithm (ADMIRE: Siemens Healthineers, Forchheim, Germany), not only are improvements in the statistical modeling applied to the raw projection data, a farther‐reaching neighborhood analysis of voxel data in the image domain is performed to attain better preservation of the CT noise texture and artifact suppression.^5^ Several recent reports incorporated some of the advantages of the advanced modeled iterative reconstruction algorithm^8^, ^9^, ^10^; however, it may be necessary to compare the results of the image analysis at the reconstruction algorithm step.

In particular, past studies have shown that using SAFIRE^11^, ^12^, ^13^ provides diagnostic quality images and reduced doses compared to FBP scans. Therefore, many clinical CT examinations try to use more advanced iterative reconstruction algorithm, such as ADMIRE.

We compared and analyzed the detectability performance corresponding to an abdominal phantom including a region of interest (ROI) according to the CT reconstruction algorithm. In this study, three types of reconstruction algorithms, namely, the FBP reconstruction method, SAFIRE, and ADMIRE, were used to evaluate the detectability performance.

## 2 METHODS AND MATERIALS

### 2.1 Experimental conditions

**Figure 1:**
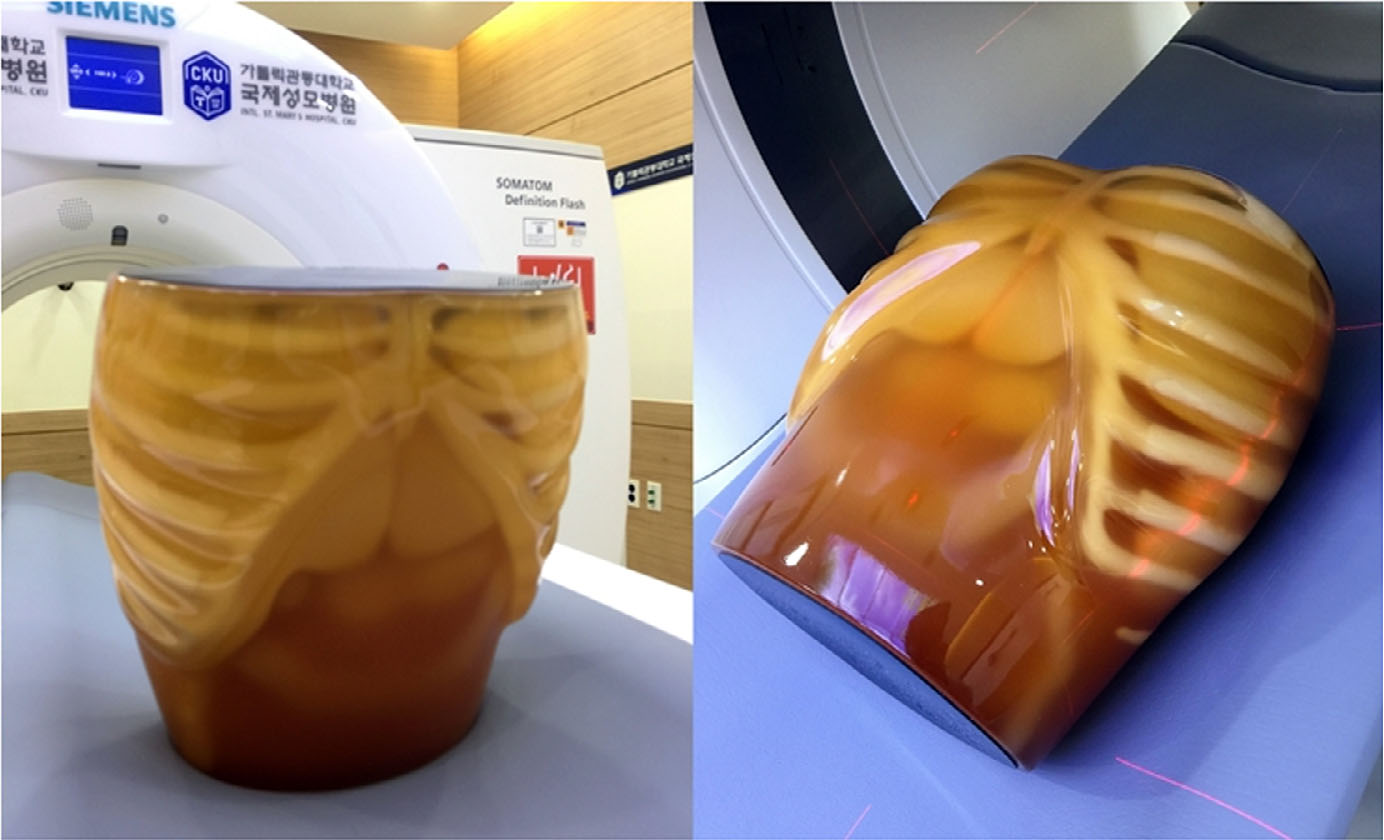
Abdominal phantom for the experiment (phantom size: 25 × 18 × 28 cm^3^). (Kyoto Kagaku, Japan)

Three types of reconstruction algorithms (FBP, SARFIRE, and ADMIRE) were used to evaluate the detectability performance using an abdominal phantom (phantom size: 25 × 18 × 28 cm^3^, see the Fig. 1). The vendor default settings for routine multi‐detector computed tomography (MDCT) abdominal scans were used, including a fixed field of view (FOV) of 300 mm, collimation of 128 × 0.6 mm, and slice thickness/increment 3.0 mm/3.0 mm. The filter kernel (herein, we used the B40f medium kernel) was selected during the iterative reconstruction, 10 investigations were performed for each case considering 9 radiation exposure parameters (80 kV/51 mAs, 80 kV/153 mAs, 80 kV/511 mAs, 100 kV/24 mAs, 100 kV/72 mAs, 100 kV/242 mAs, 120 kV/17 mAs, 120 kV/44 mAs, and 120 kV/148 mAs). The pitch was 0.6 and gantry rotation time was 0.5 s. The volume CT dose index (CTDIvol) reported by the scanner console was recorded in a DICOM dose report file after each scan. The equipment used was the SOMATOM Definition Flash CT device (Siemens Healthineers, Forchheim, Germany), and the MDCT images were reconstructed using a matrix size of 512 × 512 mm and pixel spacing (size) as 0.586 mm, an active adaptive filter, small focus size, and reading per projection (RPP) 1 × 2 z‐direction. A detailed description of the test conditions used in the reconstruction is presented in Table 1. We implemented the analysis by setting the region of interest (ROI) in MATLAB R2014a (2014a, the MathWorks Inc, USA.). The ROI size considered for the contrast‐to‐noise (CNR), coefficient of variation (COV) difference, and noise power spectrum (NPS) were 0.2 cm × 0.2 cm, and 1.5 cm × 1.5 cm, respectively. Using the statistical program (statistical package for the social sciences; SPSS version 22.0.0.0), significant differences were analyzed at 95% confidence level using a matching sample t‐test. The complete processing time at the given test conditions was less than 1 minute on a normal workstation (OS: Windows 8, CPU: 2.00 GHz, RAM: 16 GB).

**Table 1:** Scan parameters of home‐made abdominal phantom

Parameter	Dimension
Tube potential (kV)	80, 100, 120
Tube current‐time product (mAs)	51, 153, 511 (at 80 kV) 24, 72, 242 (at 100 kV) 17, 44, 148 (at 120 kV)
CT dose index (mGy)	1.01, 3, 10 [51, 153, 511 (at 80 kV)] 1, 2.99, 9.99 [24, 72, 242 (at 100 kV)] 1.18, 2.98, 10.03 [17, 44, 148 (at 120 kV)]
Slice thickness/increment (mm)	3.0/3.0
Reconstructed algorithm	FBP, SAFIRE, ADMIRE
Reconstruction kernel	B40f medium

### 2.2 The abdominal phantom

The abdominal phantom consisting of polyurethane, epoxy resin, and additional liver region was used to evaluate the image quality of MDCT. This unique anthropomorphic upper abdomen phantom allows obtaining CT images approximate to clinical data.

**Figure 2:**
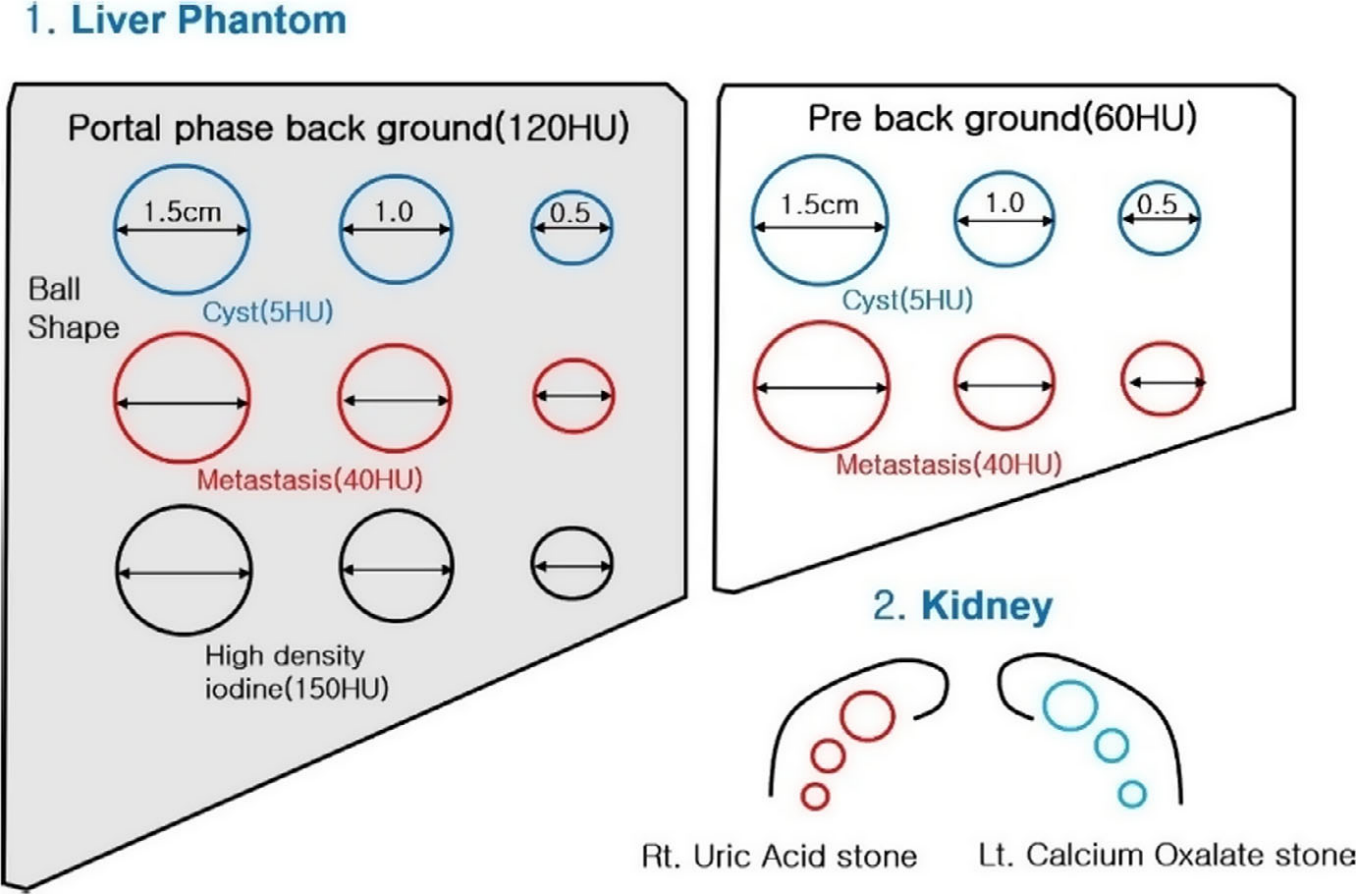
Composition of abdominal phantom made in the laboratory: Liver phantom (PH‐5 and Customized 1) Left Liver with 120 HU background — portal‐phase enhancement insert total 9 ball shaped lesion (a) cyst [5 HU] (b) Metastasis [40 HU] (c) High‐density lesion [150 HU] 2) Right liver with 60 HU background — Pre‐phase enhancement level insert total six ball‐shaped lesions (a) cyst [5 HU] (b) Metastasis [40 HU]

The elaborate anatomy of liver organs allows a multi‐dimensional approach. Figure 2 shows the abdominal phantom which is composed of the Cyst [5 HU]/Metastasis [40 HU] of the pre‐background [60 HU], and Cyst [5 HU]/Metastasis [40 HU]/High‐Density [150 HU] of Portal‐phase background [120 HU]. Each individual liver region has a particular Hounsfield number similar to that of the human liver. For the detail information of home‐made abdominal phantom, see Table 1.

**Figure 3:**
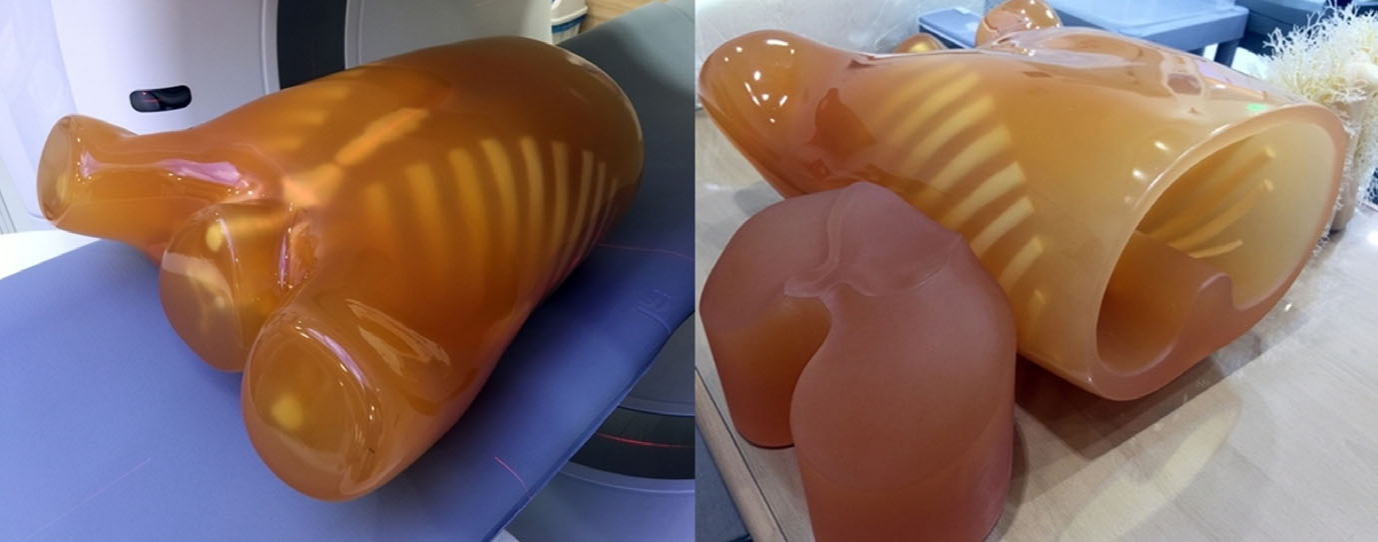
Commercialized lungman phantom (phantom size: 43 × 40 × 48 cm^3^, chest girth: 94 cm, weight: approx. 18 kg). (Kyoto Kagaku, Japan)

**Figure 4:**
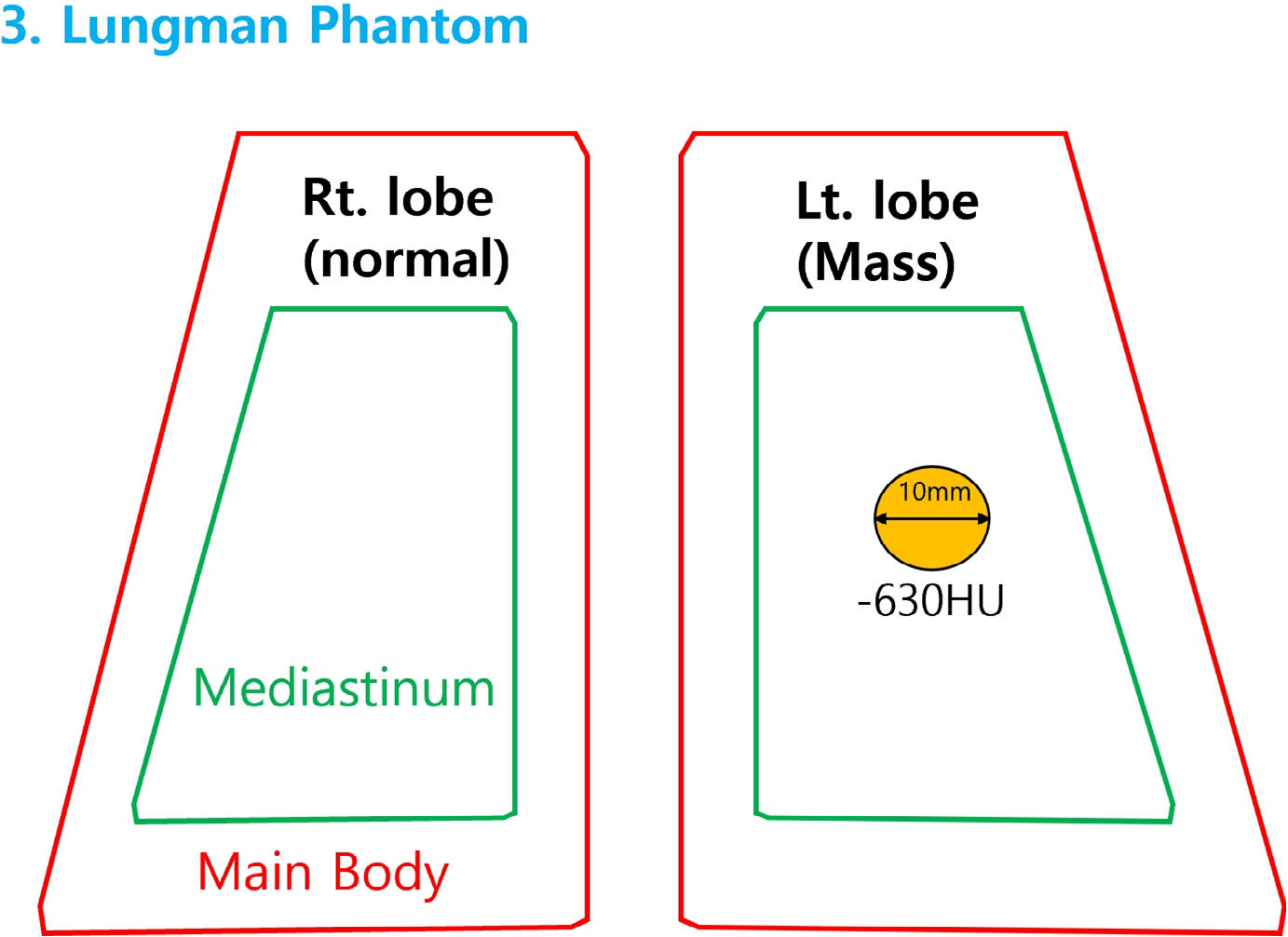
Simulated tumor (simulated tumor size: 10 mm, Approx. −630 HU, Material: Urethane form) insertion in Lt. lobe of Lungman Phantom

To compare the results of abdominal phantom study, image quality was evaluated using Lungman phantom in Fig. 3 with simulated tumor of urethane inserted. Lungman phantom can acquire CT images close to clinical data. The chest simulated tumor with a value of −630 HU of 10 mm is inserted in the left lung area and Fig. 4 shows the location of the simulated tumor. More information on the Lungman phantom is found in Table 2.

**Table 2:** Scan Parameters of Lungman phantom

Parameter	Dimension
Tube potential (kV)	100, 120
Tube current‐time product (mAs)	20, 50, 80, 110 (at 100 kV) 20, 50, 80, 110 (at 120 kV)
CT dose index (mGy)	0.82, 2.06, 3.28, 4.51 [20, 50, 80, 100 (at 100 kV)] 1.38, 3.38, 5.38, 7.41 [20, 50, 80, 100 (at 120 kV)]
Slice thickness/increment (mm)	3.0/3.0
Reconstructed algorithm	FBP, SAFIRE, ADMIRE
Reconstruction kernel	B40f medium, I40f

### 2.3 Analysis methods

For the quantitative analysis of the reconstructed images in the MDCT, we measured the contrast‐to‐noise ratio (CNR), coefficient of variation (COV), and noise power spectrum (NPS). The CNR was obtained using Eq. (1), using the ROIs (ROI_1_ and ROI_2_ in Fig. 1) and the standard deviation from the mean values of the ROIs.

(1) $$CNR=\frac{\left|{\bar{\times }}_{ROI_{1}}-{\bar{\times }}_{ROI_{2}}\right|}{\sqrt{\sigma_{ROI_{1}}^{-2}+\sigma_{ROI_{2}}^{-2}}},$$

where ${\bar{x}}_{ROI_{1}}$ and ${\bar{x}}_{ROI_{2}}$ are the mean pixel values of the predefined ROIs, respectively, and ${\bar{\sigma }}_{ROI_{1}}$ and ${\bar{\sigma }}_{ROI_{2}}$ are the standard deviations from the corresponding mean values, respectively.

The COV is defined as the ratio of the standard deviation, that is, the so‐called coefficient of dispersion as follows (Eq. (2))^14^.

(2) $$COV=\frac{\sigma }{\mu },$$

where μ is the arithmetic mean (or its absolute value, $\left|\mu \right|$) and σ is the standard deviation in the ROI. A small COV indicates better image quality because the COV reflects the noise distribution in an X‐ray image.

**Figure 5:**
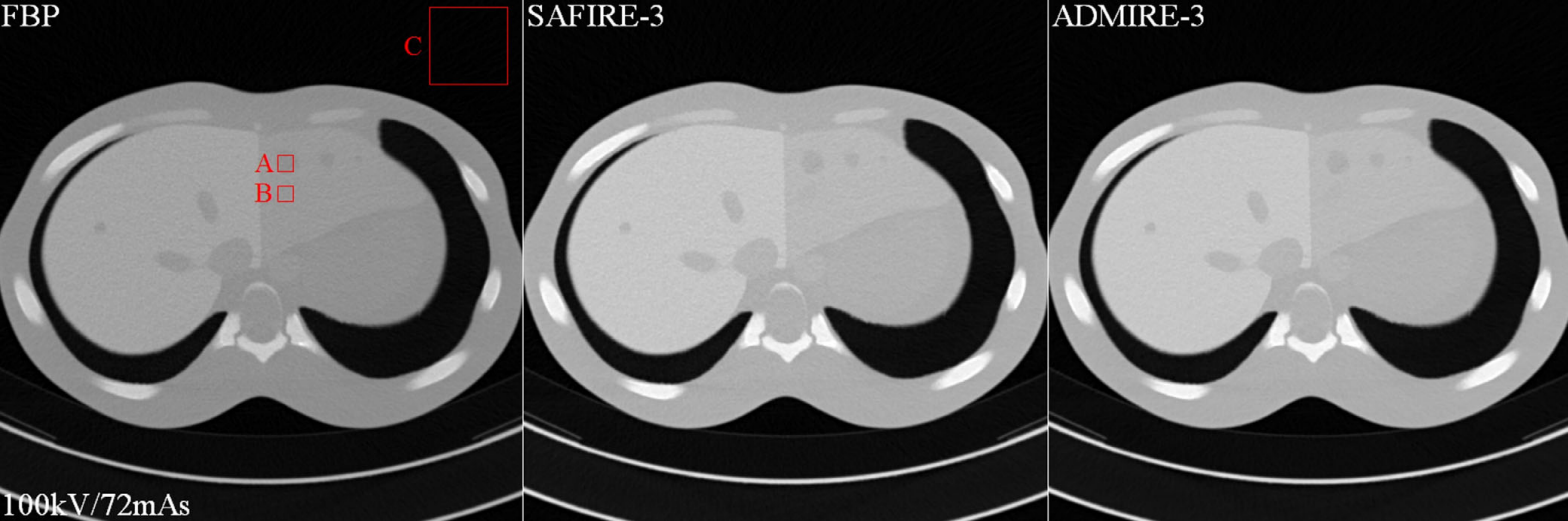
Some examples of the filtered back projection (FBP)‐based reconstructed image (left), the SAFIRE‐based reconstructed image (middle), and the ADMIRE‐based reconstructed image (right) of the abdomen phantom. Here the exposure parameters are as follows: 80 kV: 51, 153, and 511 mAs; 100 kV: 24, 72, and 242 mAs; 120 kV: 17, 44, and 148 mAs. Only three slice images (at 100 kV and 72 mAs) out of the 27 are indicated for simplicity

The NPS is expressed as the distribution of the noise frequency in the image and is defined as in Eq. (3) 15:

(3) $$NPS\left(u,v\right)=\lim_{X,Y\to \infty }\frac{1}{\mathrm{XY}}{\left|\iint_{\mathrm{XY}}\sigma \left(x,y\right)e^{-2\pi i(ux+vy)}dxdy\right|}^{2}.$$

where X and Y indicate a distance in the x‐ and the y‐directions, respectively, σ(x,y) is the difference between the average image signal and the signal at point (x,y). For the 1D NPS analysis, we obtained the reconstructed white images about 20 slices which is implemented from the same exposure conditions and reconstruction methods without object. And then sub‐ROIs of 4 and 16 parts were applied from box C in Figs. 5 and 11. Then, we calculated the two‐dimensional (2D) NPS image in Fourier domain as Eq. (3). We perform the radial averaging based on the obtained 2D NPS image for 1D NPS.

## 3 RESULTS AND DISCUSSION

**Figure 6:**
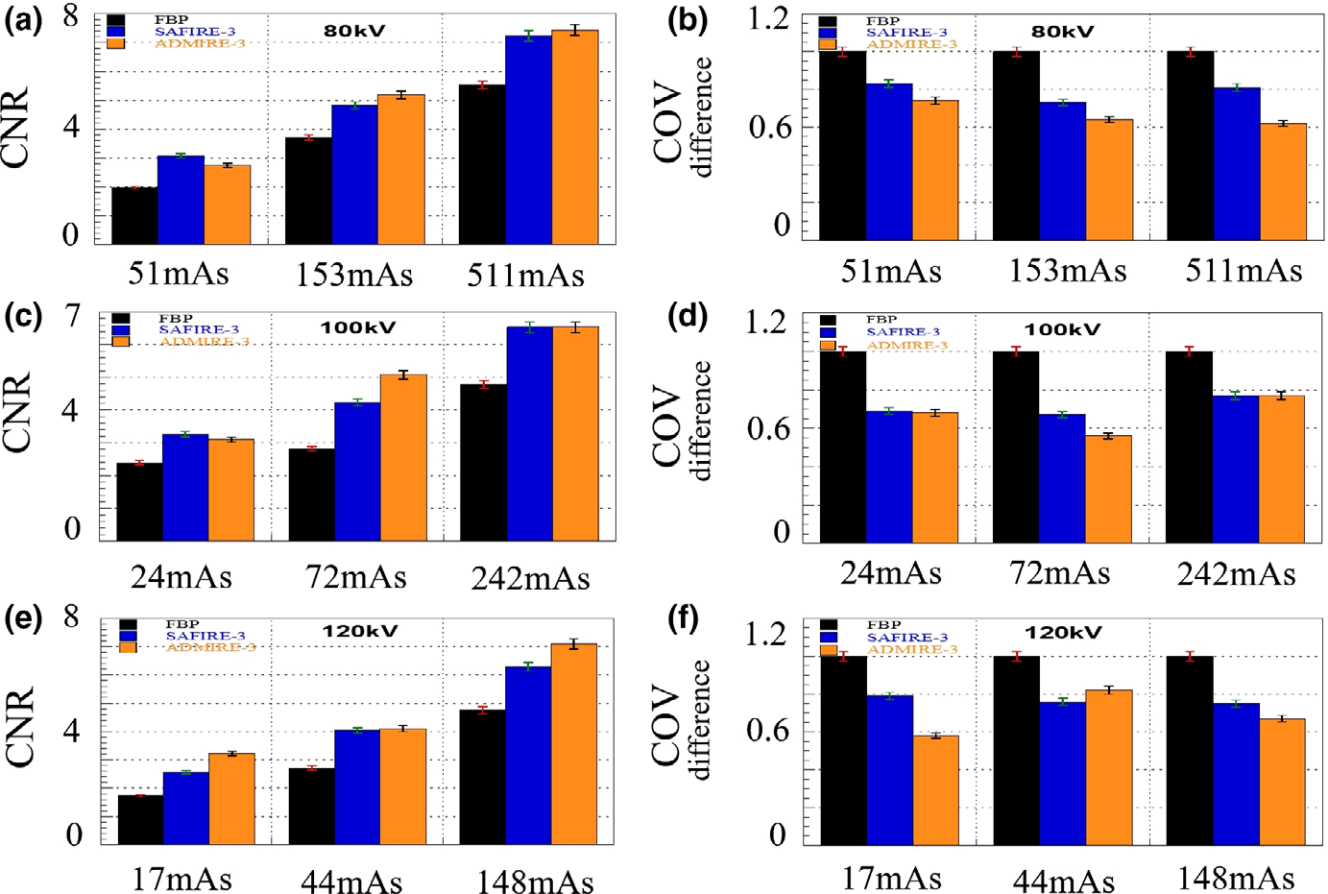
Difference in contrast‐to‐noise ratio (CNR) and coefficient of variation (COV) of images obtained using different reconstruction algorithms (FBP, SAFIRE, and ADMIRE) at different exposure parameters. (a) CNR (80 kV with 51, 153, 511 mAs), (b) COV (80 kV with 51, 153, 511 mAs), (c) CNR (100 kV with 24, 72, 242 mAs), (d) COV (100 kV with 24, 72, 242 mAs), (e) CNR (120 kV with 17, 44, 148 mAs), and (f) COV (120 kV with 17, 44, 148 mAs)

Figure 5 shows the experiment results that include the FBP reconstructed image (left), the SAFIRE reconstructed image (middle), and the ADMIRE reconstructed image (right) obtained using the abdominal phantom at the dose levels of 100 kV/72 mAs. We implemented the quantitative evaluation at the SAFIRE‐3 and ADMIRE‐3 strength. Note that the SAFIRE and ADMIRE images are quite clear to the FBP image aspect of the contrast each component and the system noise at all the exposure parameters. Box A is cyst (simulated tumor size: 1.5 cm, Approx. 5 HU, Material: polyurethane) in the abdominal phantom, Box B is pre‐background (Approx. 60 HU) in the abdominal phantom, and Box C is air (Approx.1000 HU). For quantitative evaluation of the three reconstruction algorithms, Fig. 6 shows the CNR and COV difference values indicated by the boxes A and B in Fig. 5 at all exposure parameters. The CNR and COV difference results at all exposure parameters. The COV measured the ROI indicated the box B. Here, we used the reconstruction algorithms of the FBP, SAFIRE‐3, and ADMIRE‐3 strength, where that the characteristic of the ADMIRE‐3 strength was noted.

The noise characteristic of ADMIRE reconstructed image was higher than those of FBP and SAFIRE reconstructed images. The CNR values of the SAFIRE and ADMIRE images were much higher than those of the FBP images, and the difference COV in the ADMIRE images was approximately 1.2 times lower than the corresponding values of the SAFIRE images. The result values have a validity of 95% (*P* < 0.05).

**Figure 7:**
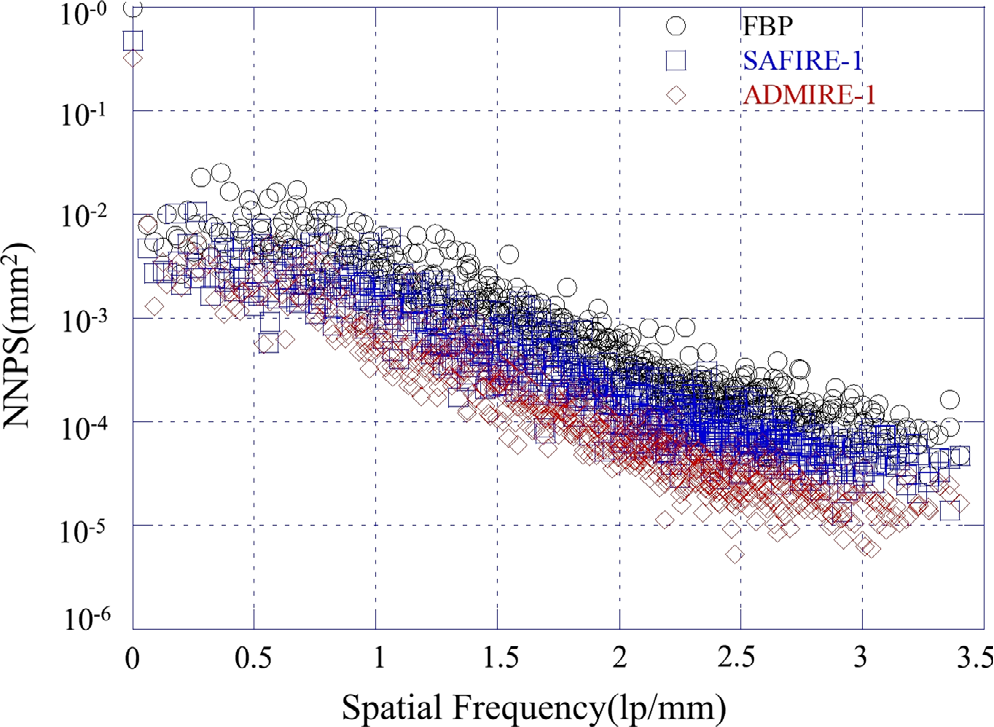
The measured 1D NPS curves indicated by the box C in Fig. 5 using only strength 1. (The reconstructed images of the FBP, the SAFIRE‐1, and the ADMIRE‐1 at the condition of the 100 kV/72 mAs)

**Figure 8:**
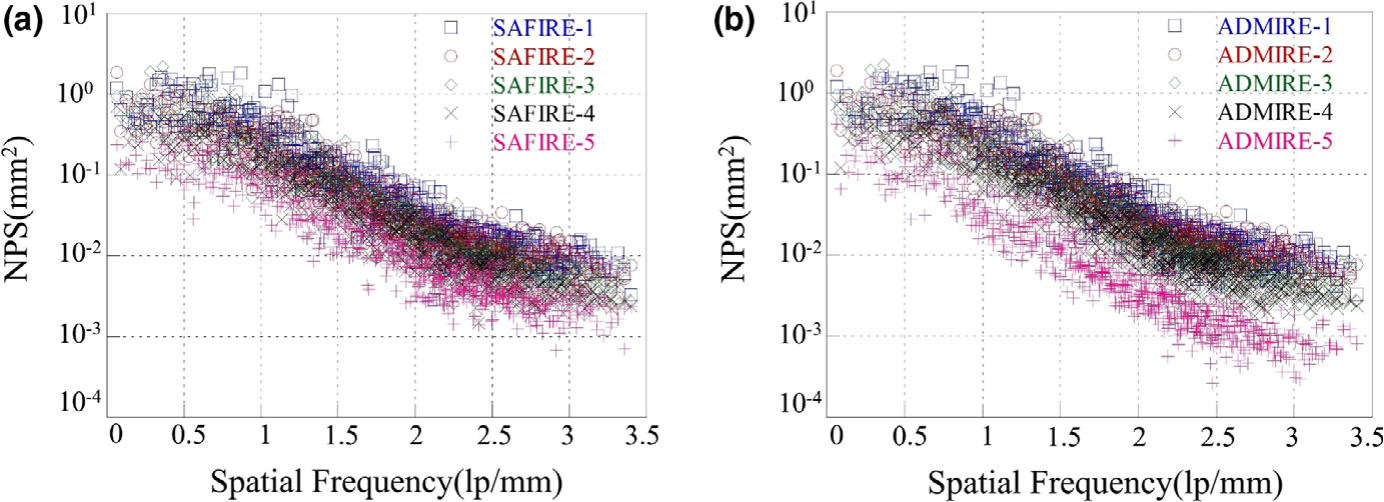
The combined 1D NPS curves indicated by the box C in Fig. 5 using from 1 to 5 strength for the SAFIRE and ADMIRE reconstruction algorithm. (a) The reconstructed images of the SAFIRE‐1 to 5 at the condition of the 100 kV/72 mAs) (b) the reconstructed images of the ADMIRE‐1 to 5 at the condition of the 100 kV/72 mAs)

Figure 7 shows the measured 1D NPS curves indicated by the box C in Fig. 5 for the reconstructed images of the FBP, the SAFIRE‐1, and the ADMIRE‐1 at the condition of the 100 kV/72 mAs. Note that the 1D NPS value of the ADMIRE‐5 was about 0.1 times lower than the NPS values of the other reconstructed images at all spatial frequency. Figure 8 shows the noise quality of the reconstructed images for each strength in (a) the SAFIRE and (b) the ADMIRE algorithm. According to the results, the noise quality of the reconstructed images of the SAFIRE and ADMIRE algorithms, these values of NPS decreases from 1 to 5 strength, sequentially.

**Figure 9:**
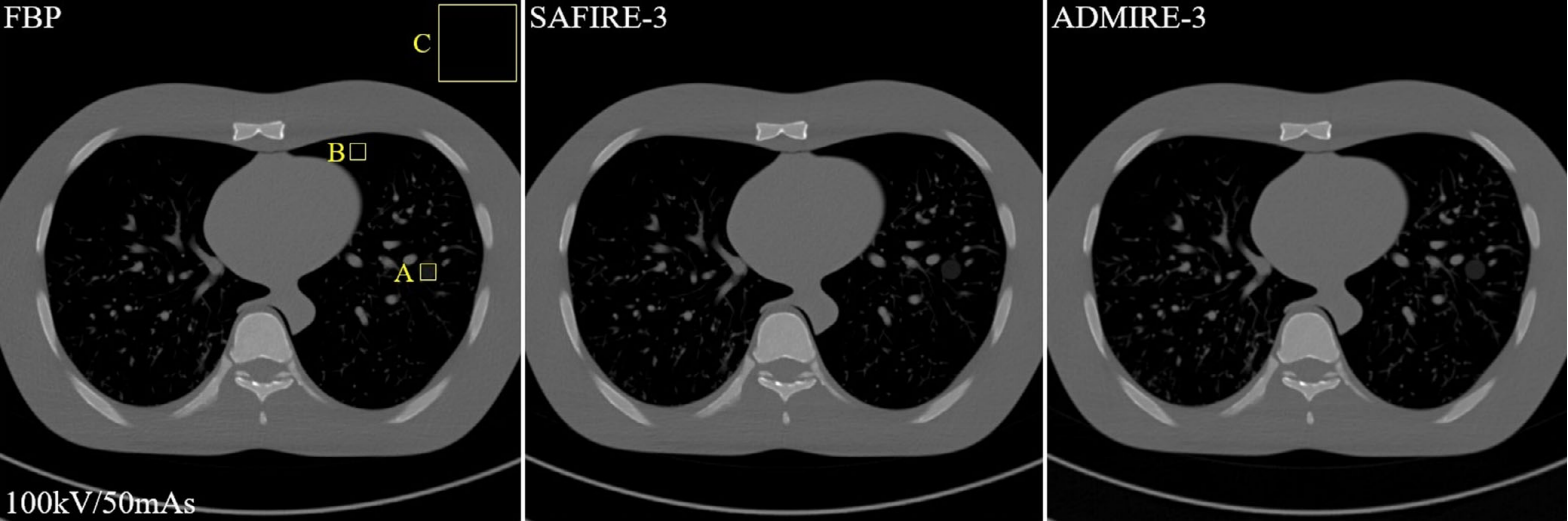
Complete sets of the FBP‐based reconstructed image of a phantom chest, the SAFIRE‐based reconstructed image, and the ADMIRE‐based reconstructed image. The phantom has a small‐ball material in box A. Here, the SAFIRE and ADMIRE also used strength‐3. Box A is simulated tumor (simulated tumor size: 10 mm, Approx. −630 HU, Material: Urethane form) in the Lungman phantom, Box B is mediastinum (approx. −1000 HU) in the Lungman phantom and Box C is air (approx. −1000 HU)

**Figure 10:**
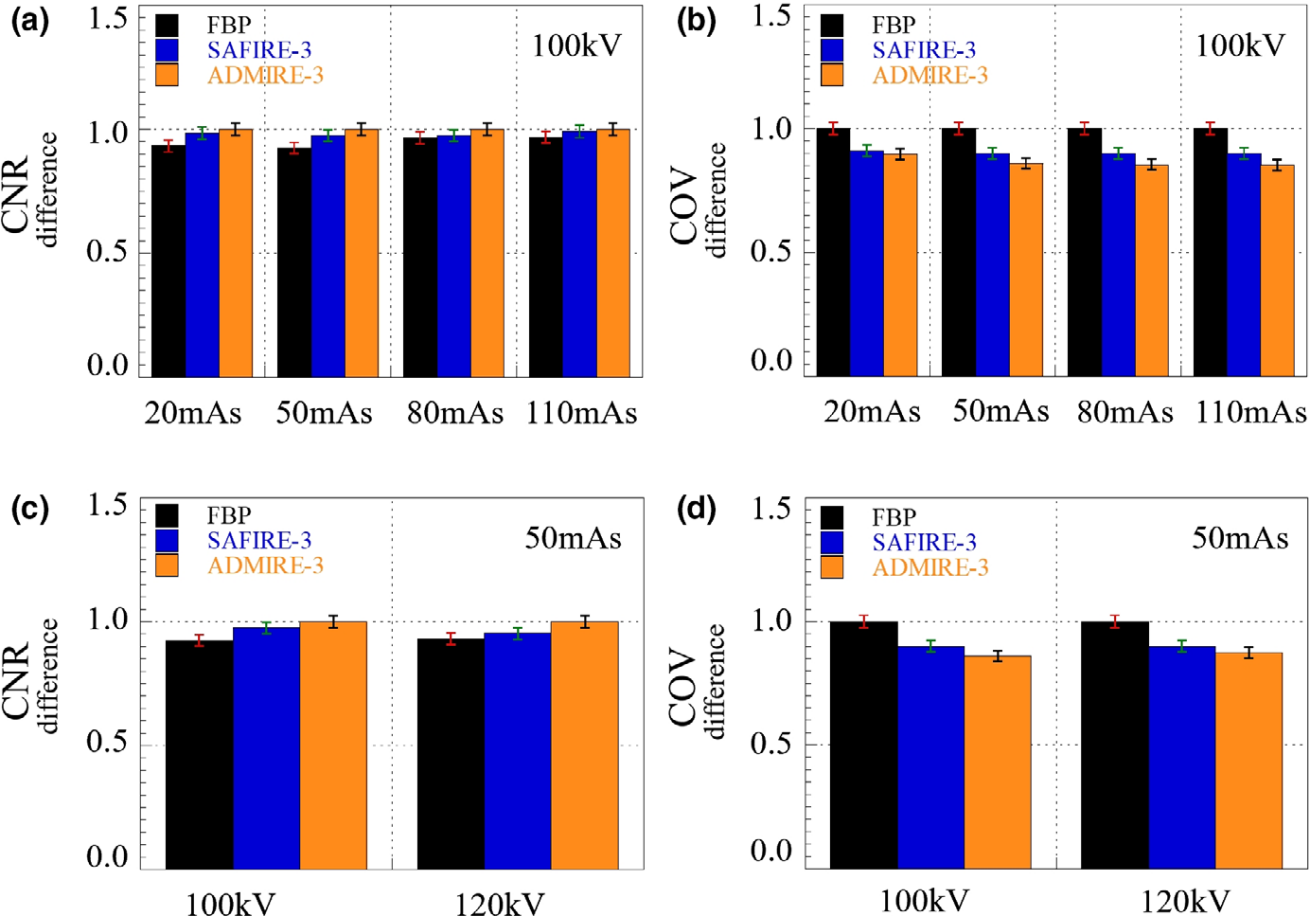
Measured differences contrast‐to‐noise ratio (CNR) and coefficient of variation (COV) values from the z = 38th slice images indicated by box A and B in Fig. 9 for the FBP, SAFIRE, and ADMIRE cases. For all conditions, the ADMIRE‐based reconstructed images demonstrate much better quality than the SAFIRE‐ and FBP‐based reconstructed images. (a) CNR difference (100 kV with 20, 50, 80, and 110 mAs), (b) COV difference (100 kV with 20, 50, 80, and 110 mAs), (c) CNR difference (50 mAs with 100 and 120 kV), (d) COV difference (50 mAs, with 100 and 120 kV)

Figure 9 shows the complete sets of the FBP‐based reconstructed image of a chest region in the abdominal phantom, the SAFIRE‐based reconstructed image, and the ADMIRE‐based reconstructed image. Box A is simulated tumor (simulated tumor size: 10 mm, Approx. −630 HU, Material: Urethane form) in the Lungman phantom, Box B is mediastinum (approx. −1000 HU) in the Lungman phantom, and Box C is air (approx. −1000 HU). The phantom has a small‐ball material in box A. Here, the SAFIRE and ADMIRE applied also used mode 3. Figure 10 shows the measured CNR difference and COV difference values from the z = 38th slice images indicated by boxes A and B in Fig. 9 for the FBP, SAFIRE, and ADMIRE cases. For all conditions, the ADMIRE‐based reconstructed images demonstrate much better quality than the SAFIRE‐ and FBP‐based reconstructed images.

**Figure 11:**
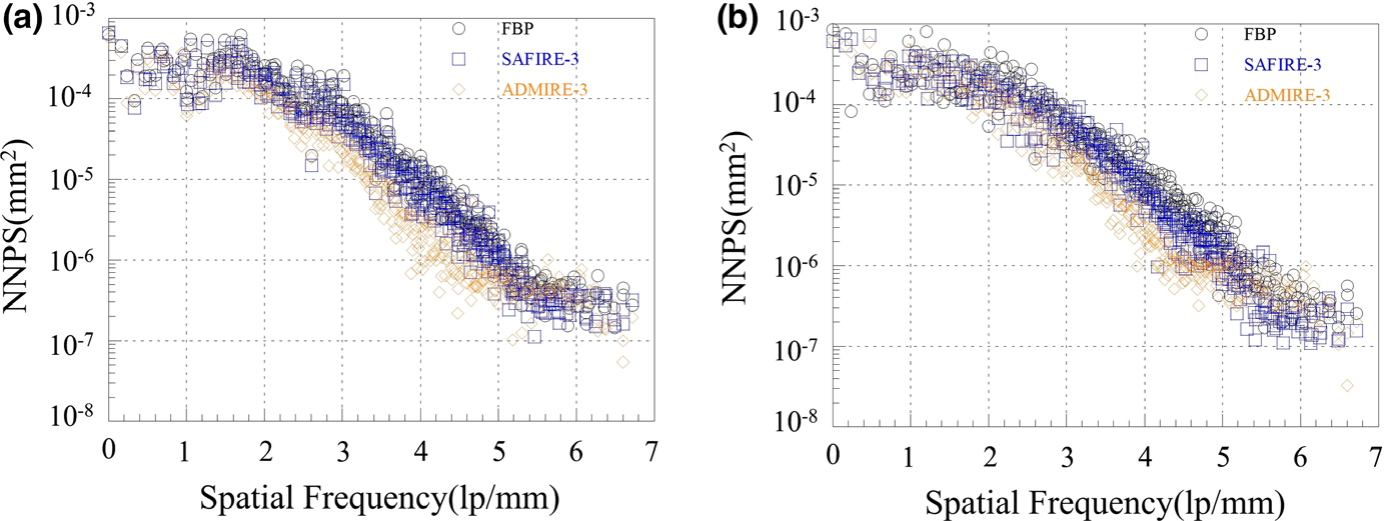
Resultant 1D NPS curves for the FBP‐, SAFIRE‐, and ADMIRE‐based reconstructed image cases indicated by the Box C in Fig. 9 using only strength‐3. The NPS quality of the ADMIRE image was much improved in the conditions of the (a) 100 kV/50 mAs and (b) 120 kV/50 mAs

Difference values from the z = 38th slice images indicated by boxes A and B in Fig. 9 for the FBP, SAFIRE, and ADMIRE cases. For all conditions, the ADMIRE‐based reconstructed images demonstrate much better quality than the SAFIRE‐ and FBP‐based reconstructed images. Figure 11 shows the resultant 1D NPS curves that gradually decrease as the spatial frequency increases from box C in Fig. 9. The smaller the NPS value while the spatial frequency increases, the better noise characteristic of image. The NPS quality of the ADMIRE image is at spatial frequency.

**Figure 12:**
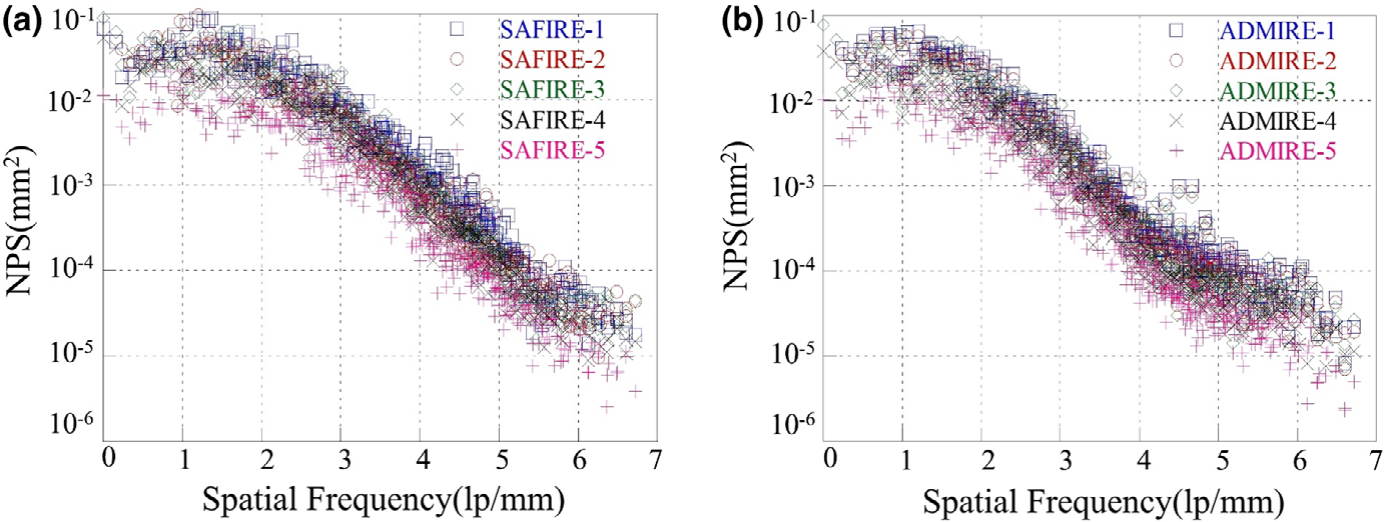
Resultant 1D NPS curves for the SAFIRE, and ADMIRE‐based reconstructed image cases indicated by the box C in Fig. 9. (a) The reconstructed images of the SAFIRE‐1 to 5 at the condition of the 120 kV/50 mAs, (b) the reconstructed images of the ADMIRE‐1 to 5 at the same condition of the (a)

Frequencies of over about 1.0 lp/mm were improved, compared to those of the SAFIRE‐based reconstructed image, and the FBP‐based reconstructed image at the exposure conditions of 100 kV/50 mAs and 120 kV/50 mAs. Figure 12 shows also the noise quality of the Lungman phantom’s reconstructed images for each mode in (a) the SAFIRE and (b) the ADMIRE algorithm at the exposure condition of the 120 kV/50 mAs. Based on our results, we verified the better noise characteristic of ADMIRE method, compared to that of SAFIRE and FBP methods. These features are in line with ADMIRE’s goal of the separation of noise from real anatomic structures in the image and there are also consistent with the results of the following papers those are investigated in relation to these features between ADMIRE and SAFIRE.^16^, ^17^ These papers are investigated for noise characteristic and edge sharpness at the conditions of each clinical case (i.e., here, they are researched in pulmonary and abdomen) using the quantitative evaluation including receiver operating characteristic (ROC) curve, area under the ROC curve (AUC), and image noise considering the ROI. In addition, our results are meaningful so that the noise characteristics between both of reconstruction algorithms for various exposure conditions and body parts (e.g., we designed the liver and lung phantoms) were confirmed by quantitative statistical indicators such as CNR, COV, and NPS. In this regard, the results of the studies on ADMIRE, SAFIRE, and FBP can be supported to effectively control CT image quality and dose reduction in the abdomen and lungs.

## 4 CONCLUSION

In this work, we investigated the image performance of ADMIRE‐based reconstructed images using quantitative evaluation methods, compared to that of FBP‐ and SAFIRE‐based reconstructed images. From the comparative analysis of the abdominal phantom low‐contrast resolution differences for each CT exposure parameters, and through a variety of analysis methods, it was determined that ADMIRE obtained better results than SAFIRE and FBP. The CNR value for the ADMIRE‐based reconstructed images was approximately 1.2 times higher than those for the other reconstructed images using the hand‐made abdominal phantom. The ADMIRE image resultants of COV difference with normalized COV with FBP show the lower value than COV difference value of FBP and SAFIRE images. The results of the commercialized Lungman phantom indicated almost same tendency, compared to those of the hand‐made abdominal phantom. According to our results, the ADMIRE can provide more clear observation aspects of contrast and low‐contrast resolution, compared to FBP and SAFIRE and facilitate the achievement of an accurate diagnosis.

## CONFLICT OF INTEREST

There is no conflict of interest for all contents in this paper.
